# Effects of three flavonoids on the metabolism of lenvatinib

**DOI:** 10.3389/fphar.2024.1438259

**Published:** 2024-08-20

**Authors:** Jinzhao Yang, Jie Chen, Qingqing Li, Ren-ai Xu, Xiaohai Chen

**Affiliations:** ^1^ Wenzhou People’s Hospital, The Third Clinical Institute Affiliated to Wenzhou Medical University, Wenzhou, Zhejiang, China; ^2^ The First Affiliated Hospital of Wenzhou Medical University, Wenzhou, Zhejiang, China; ^3^ The Third Affiliated Hospital of Wenzhou Medical University, Wenzhou, Zhejiang, China

**Keywords:** hepatocellular carcinoma, lenvatinib, flavonoids, luteolin, DDIs

## Abstract

Lenvatinib is a first-line therapy for the treatment of hepatocellular carcinoma (HCC), an active multi-target tyrosine kinase inhibitor (TKI). The interaction between Traditional Chinese Medicine (TCM) and chemicals has increasingly become a research hotspot. The objective of this study was to pinpoint the effects of three flavonoids on the metabolism of lenvatinib. Enzyme reaction system was established and optimized *in vitro*, and *in vivo* experiments were conducted in Sprague-Dawley (SD) rats, where the analytes were detected by ultra performance liquid chromatography tandem mass spectrometry (UPLC-MS/MS). We found that among three flavonoids, luteolin and myricetin had strong inhibitory effects on lenvatinib metabolism, with half-maximal inhibitory concentration (IC_50_) values of 11.36 ± 0.46 µM and 11.21 ± 0.81 µM in rat liver microsomes (RLM), respectively, and 6.89 ± 0.43 µM and 12.32 ± 1.21 µM in human liver microsomes (HLM), respectively. In Sprague-Dawley rats, the combined administration of lenvatinib and luteolin obviously expanded the exposure to lenvatinib; however, co-administered with myricetin did not have any changes, which may be due to the poor bioavailability of myricetin *in vivo*. Furthermore, the inhibitory type of luteolin on lenvatinib showed an un-competitive in RLM and a mixed in HLM. Collectively, flavonoids with liver protection, especially luteolin, may inhibit lenvatinib metabolism *in vitro* and *in vivo*.

## 1 Introduction

Liver disease remains one of the leading causes of death worldwide (Qian et al., 2021). It is estimated that approximately two million people around the world die from this disease (Mokdad et al., 2014), of which liver cancer ranks third among the global causes of cancer death (Rumgay et al., 2022), while hepatocellular carcinoma (HCC) is the most common form of liver cancer and the second leading cause of death from malignant tumors in the world (Mazzanti et al., 2016). This is mainly due to the fact that most people do not have symptoms in the initial stages of HCC, until the terminal stage, when liver transplantation is not possible (Ogunwobi et al., 2019; Buttell and Qiu, 2023), ultimately leading to a continuous increase in incidence and a 5-year survival rate of less than 20% (Chou et al., 2014).

Lenvatinib is an oral small molecule tyrosine kinase inhibitor (TKI) that selectively inhibits vascular endothelial growth factor receptors 1–3 (VEGFR 1–3), fibroblast growth factor receptors (FGFR), platelet-derived growth factor receptor α (PDGFRα), and proto-oncogenes kinase inhibitor (KIT) (Matsui et al., 2008; Ikuta et al., 2009). At present, the United States Food and Drug Administration (FDA) has approved it as a first-line treatment of unresectable HCC (Nair et al., 2021), and its therapeutic effect is not inferior to that of sorafenib (Kudo, 2018). The major metabolic product of lenvatinib is *O*-desmethyl lenvatinib (M1), and current studies have shown that CYP1A1, CYP1A2, CYP2B6, and CYP3A4 are the most efficient enzymes for the formation of these metabolites (Vavrová et al., 2022). Nevertheless, lenvatinib often has adverse reactions such as hepatotoxicity (Furuse et al., 2023), hypertension, hand-foot syndrome, and thrombocytopenia during treatment (Suyama and Iwase, 2018). Drug adverse reactions are one of the main causes of morbidity and mortality in the clinic every year (Wu et al., 2014), where drug interaction accounts for 30% of all adverse drug events (Becker et al., 2007).

Drug-drug interactions is a major problem in clinical practice and has been recognized as one of the primary threats to public health (Sennesael et al., 2018; Hoel et al., 2021; Ye et al., 2021; Zerah et al., 2021; Bruggemann et al., 2022). Traditional Chinese Medicine (TCM) is frequently used in combination with chemotherapeutic drugs (Hu et al., 2005). In addition to flavonoids, alkaloids, terpenoids, and others have been proven to have good anti-tumor effects (Zhao et al., 2023). Among them, flavonoids are a type of polyphenols widely found in fruits, vegetables, beer, and other common foods (Santes-Palacios et al., 2020). Research by Aune et al. indicates that its intake is closely related to reducing the incidence of cancer (Aune et al., 2017). For instance, luteolin can improve liver lesions by inhibiting inflammatory factors, alleviating oxidative stress, inducing liver cancer cell apoptosis and autophagy (Yao et al., 2023); myricetin can suppress the progression of hepatocellular carcinoma by decreasing the regulation of YAP expression (Li et al., 2019); fisetin can protect the liver by increasing GSH and reducing inflammatory mediators and CYP2E1 (Ugan et al., 2023). However, the interaction between flavonoids and lenvatinib has rarely been reported.

Therefore, in this study, we systematically selected luteolin (Yao et al., 2023), myricetin (Xu et al., 2020), and fisetin (Ugan et al., 2023), three kinds of flavonoids with live protective effects, to explore the changes of lenvatinib at present of them. First, *in vitro*, we used rat liver microsomes (RLM) and human liver microsomes (HLM) to investigate their effects on lenvatinib metabolism and the corresponding potential mechanisms. Subsequently, *in vivo*, Sprague-Dawley (SD) rats were applied to discover changes in the pharmacokinetic parameters of lenvatinib by luteolin and myricetin. The results may provide certain data to support the personalized precision treatment of lenvatinib in clinical practice.

## 2 Materials and methods

### 2.1 Chemicals and reagents

Lenvatinib, *O*-desmethyl lenvatinib (M1), internal standard (IS, regorafenib), melatonin, 6-hydroxy melatonin, bupropion, hydroxy bupropion, midazolam and 1′-hydroxy midazolam were purchased from Beijing Sunflower Technology Development Co., Ltd (Beijing, China). Luteolin, myricetin and fisetin were provided by Shanghai Canspec Scientific Instruments Co., Ltd. (Shanghai, China). Reduced nicotinamide adenine dinucleotide phosphate (NADPH) was procured from Shanghai Aladdin Biochemical Technology Co., Ltd. (Shanghai, China). HLM (mixed gender, pool of 50 donors) was from iPhase Pharmaceutical Services Co., Ltd. (Jiangsu, China), while RLM was prepared by our team. All solvents and reagents included in this study were above of analytical grade.

### 2.2 Detection condition of UPLC-MS/MS

A Waters Acquity ultra performance liquid chromatography tandem mass spectrometry (UPLC-MS/MS) system was used for the quantitative analysis of the analytes. Chromatographic separation of lenvatinib, M1 (the main metabolite of lenvatinib), and IS was carried out by an Acquity BEH C18 column (2.1 mm × 50 mm, 1.7 μm; Milford, MA, United States) at a temperature of 40°C. The mobile phase was composed of 0.1% formic acid (A) and acetonitrile (B), with a gradient elution at a flow rate of 0.40 mL/min for 2 min. Mass spectrometry information of the analytes were obtained by a Waters Xevo TQ-S triple quadrupole tandem mass spectrometer (Milford, MA, United States) with multiple reaction monitoring (MRM) in positive mode. Furthermore, the main operating parameters of the mass spectrometers for lenvatinib, M1, IS and specific CYP isoform probes were summarized in Table 1.

**TABLE 1:** Ion transitions and the main operating parameters for lenvatinib, M1, regorafenib (IS) and specific CYP isoform probes of the mass spectrometer.

Analytes	Ion transition (*m/z* to *m/z*)	Cone (V)	Collision (eV)
Lenvatinib	427.20 → 369.87	30	28
M1 (*O*-desmethyl lenvatinib)	413.89 → 339.85	10	28
Regorafenib (IS)	483.00 → 269.97	20	30
6-hydroxy melatonin	248.94 → 190.00	10	10
hydroxy bupropion	256.01 → 237.99	20	10
1′-hydroxy midazolam	341.96 → 324.03	50	20

### 2.3 Enzyme preparation of RLM

The enzyme preparation of RLM was referred to previously reported literature (Wang et al., 2015). The main processes were as follows: the rat liver was weighted on balance and homogenized according to the ratio of 1 g plus 2.5 mL pre-cooled PBS-0.25 mM sucrose buffer. The homogenates were then centrifuged at 11,000 rpm for 15 min at 4°C, and after repeated centrifugation of the supernatants, the supernatant was centrifuged at 75,600 × *g* at 4°C for 2 h. Next, the precipitations were mixed with pre-cooled PBS buffer in a 1:3 ratio to obtain RLM. Finally, a BCA protein assay kit (Thermo Scientific) was used to determine the protein concentration. Absorbance was measured at 562 nm, and the results showed that the average concentration was 17.58 ± 0.63 mg/mL.

### 2.4 Enzyme reaction system

To determine the enzymatic kinetic parameter (the Michaelis constant, K_m_) of lenvatinib in RLM and HLM, we established a 200 μL enzyme reaction system, which included 2–100 μM lenvatinib, 0.3 mg/mL RLM or HLM, 0.1 M Tris-HCl, and 1 mM NADPH. In this study, the K_m_ of specific CYP isoform probes, including melatonin (CYP1A2), bupropion (CYP2B6) and midazolam (CYP3A4), were determined by a mixed method. The mixed probe substrates were added to an incubation mixture containing 0.3 mg/mL RLM, 0.1 M PBS, and 1 mM NADPH. First, the mixture without NADPH was pre-incubated at 37°C for 5 min, and then 10 μL NADPH was added to start the reaction. After incubation for 30 min, the samples were immediately freezed to −80°C to terminate the reaction. About 20 min later, 300 μL acetonitrile and 10 μL regorafenib (200 ng/mL) as IS were added to precipitate the protein. When the samples were fully vortexed for 2 min and completely mixed, the mixture was centrifuged for 10 min at 13,000 rpm. In the end, each supernatant was detected and analyzed by UPLC-MS/MS.

To explore the ability of three flavonoids to inhibit lenvatinib metabolism *in vitro*, we measured the half-maximal inhibitory concentration (IC_50_) of luteolin, myricetin, and fisetin, respectively. The 200 μL incubation system consisted of Tris-HCl (0.1 M), RLM or HLM (0.3 mg/mL), NADPH (1 mM), lenvatinib (23.5 μM in RLM or 16.9 μM in HLM) and three flavonoids (0, 0.01, 0.1, 1, 10, 25, 50 and 100 μM). To evaluate the effects of three flavonoids on CYP1A2, CYP2B6 and CYP3A4 activity in RLM, the specific CYP isoform probe mixture was incubated with a single inhibitor and the specific CYP isoform probe was used at a concentration equal to or slightly below its corresponding K_m_ value (Dinger et al., 2014). A similar incubation system was established including PBS (0.1 M), RLM (0.3 mg/mL), NADPH (1 mM), mixed probes (30.0 μM melatonin, 8.0 μM bupropion and 1.5 μM midazolam) and three flavonoids (0, 0.01, 0.1, 1, 10, 25, 50 and 100 μM). Subsequently, on the basis of the IC_50_ values and the data from animal experiments, we deeply studied the potential mechanism of interaction between lenvatinib and luteolin. In the 200 μL mixture, the concentration of lenvatinib was set to 5.88, 11.75, 23.50, 47.00 μM in RLM and 4.23, 8.45, 16.90, 33.80 μM in HLM (according to the corresponding K_m_ value), and the concentration of luteolin was 0, 2.94, 5.89, 11.77 μM in RLM and 0, 3.44, 6.87, 13.74 μM in HLM (according to the corresponding IC_50_ value). Finally, the samples were processed as mentioned above.

### 2.5 Metabolic stability

Using the UPLC-MS/MS technique, metabolic stability tests were carried out to monitor any discernible drop in lenvatinib concentration in the RLM matrix (Shang et al., 2021). 1.0 μM lenvatinib was incubated with 0.3 mg/mL RLM and 1 mM NADPH in 0.1 M Tris-HCl buffer until a final volume of 0.2 mL was reached. NADPH was introduced following a 5 min pre-incubation period, and the reaction was stopped at various intervals of 0, 5, 10, 45, 60, and 90 min. Similarly, three flavonoids were added to the above incubation system to study the effects of the three flavonoids on the metabolic stability of lenvatinib. The post-treatment method was same as the above enzyme reaction. The metabolic stability curve for lenvatinib was constructed from the obtained data.

### 2.6 Animal experiments

In this study, healthy male Sprague-Dawley rats (200 ± 10 g) were obtained from the Animal Experimental Center of the First Affiliated Hospital of Wenzhou Medical University (Zhejiang, China). Experiment animals were cared as required by the National Research Council Guidelines for the Care and Use of Laboratory Animals. Moreover, the research protocol was in accordance with the ARRIVE guidelines and was approved by the Experimental Animal Ethics Committee of the First Affiliated Hospital of Wenzhou Medical University (Ethics approval number: WYYY-IACUC-AEC-2024–014).

Fifteen SD rats were randomly divided into three groups (n = 5): Group A, lenvatinib (1.2 mg/kg, p. o.) (Cui et al., 2021); Group B, lenvatinib (1.2 mg/kg, p. o.) + luteolin (30 mg/kg, p. o.) (Chen et al., 2010); Group C, lenvatinib (1.2 mg/kg, p. o.) + myricetin (50 mg/kg, p. o.) (Lan et al., 2017). Three drugs were suspended in 0.5% carboxymethylcellulose sodium salt (CMC-Na) solution, respectively, prepared when used. Before the formal experiment, SD rats fasted for 12 h to avoid the effect of food intake on drug absorption, but were free to drink water. At the beginning of the experiment, group A was given equal volumes of 0.5% CMC-Na solution, while group B and C were received luteolin and myricetin, respectively. After 30 min, all three groups were gavaged with a single dose of 1.2 mg/kg lenvatinib. At 1, 1.5, 2, 3, 4, 6, 8, 12, and 24 h after lenvatinib administration, blood samples were collected from the tail vein into a 1.5 mL EP tube treated with heparin, respectively. The 50 μL plasma supernatant was precisely extracted and put into a new 1.5 mL EP tube after centrifugation at 8,000 rpm for 10 min. 150 μL acetonitrile and 10 μL IS working solution (200 ng/mL) were added and fully vortexed, then centrifuged for 10 min at 13,000 rpm. Finally, the liquid supernatant was taken to UPLC-MS/MS to detect the concentration of the analytes.

### 2.7 Statistical analysis

The experimental data were expressed as mean ± standard deviation (mean ± S.D.). Through GraphPad Prism 9.5 software, the Michaelis-Menten curves of lenvatinb in RLM and HLM were drawn based on the Michaelis-Menten analysis curve fitting program in nonlinear regression analysis, and the log(inhibitor) vs normalized response mode was applied to acquire the corresponding IC_50_ curve diagram. The Lineweaver-Burk plot was got by the Lineweaver-Burk double reciprocal mode, and its subsidiary plot was drawn on the slope (K_m_/V_max_ vs. inhibitor concentration) and *y*-intercept (1/V_max_ vs. inhibitor concentration) of the drawn line. Besides, the mean plasma concentration-time curve of lenvatinib was plotted based on the pharmacokinetic data. Drug and Statistics (DAS) software (version 3.0 software, Mathematical Pharmacology Professional Committee of China, Shanghai, China) with non-compartment model analyzes was used to obtain the corresponding pharmacokinetic parameters. SPSS (version 24.0; SPSS Inc., Chicago, IL, United States of America) with one-way ANOVA was used to compare the difference between the combination and the single group, respectively. *p <* 0.05, it was considered statistically significant.

## 3 Results

### 3.1 UPLC-MS/MS method for the determination of lenvatinib and M1

**FIGURE 1:**
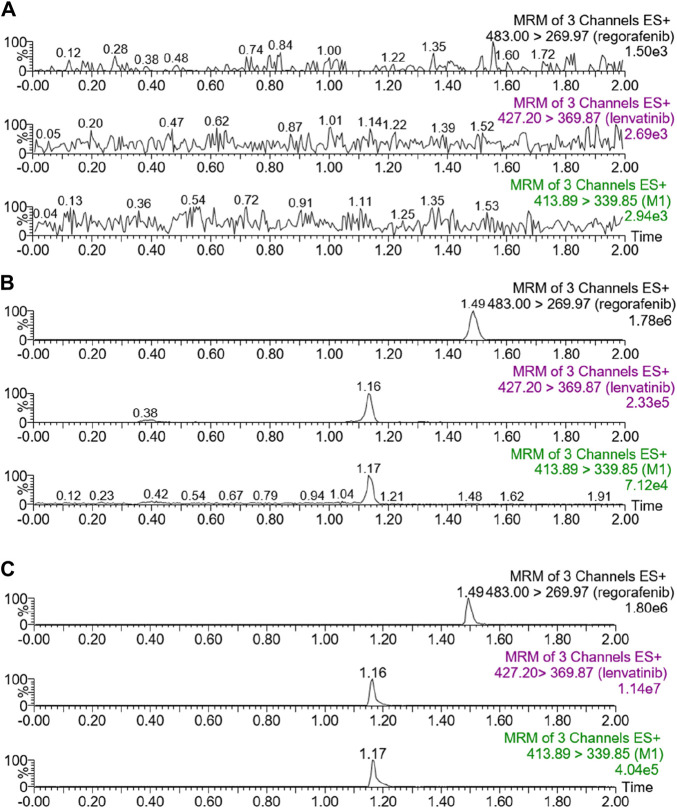
UPLC-MS/MS chromatograms of lenvatinib, M1 and regorafenib (IS). **(A)** Blank rat plasma. **(B)** Blank rat plasma added with analytes at LLOQ. **(C)** Rat plasma samples from animal experiments.

The chromatograms in Figure 1 showed that within 2 min of elution time, the retention times of lenvatinib, M1, and IS were 1.16 min, 1.17 min, and 1.49 min, respectively, could be well separated without interference from endogenous substances. Furthermore, lenvatinib and M1 had good linear relationships in the range of 2–500 ng/mL and 2–20 ng/mL, respectively, both with correlation coefficients greater than 0.99. The lower limit of quantification (LLOQ) of lenvatinib and its main metabolite was 2 ng/mL.

### 3.2 Luteolin and myricetin both have strong inhibitory effects on the metabolism of lenvatinib *in vitro*

**FIGURE 2:**
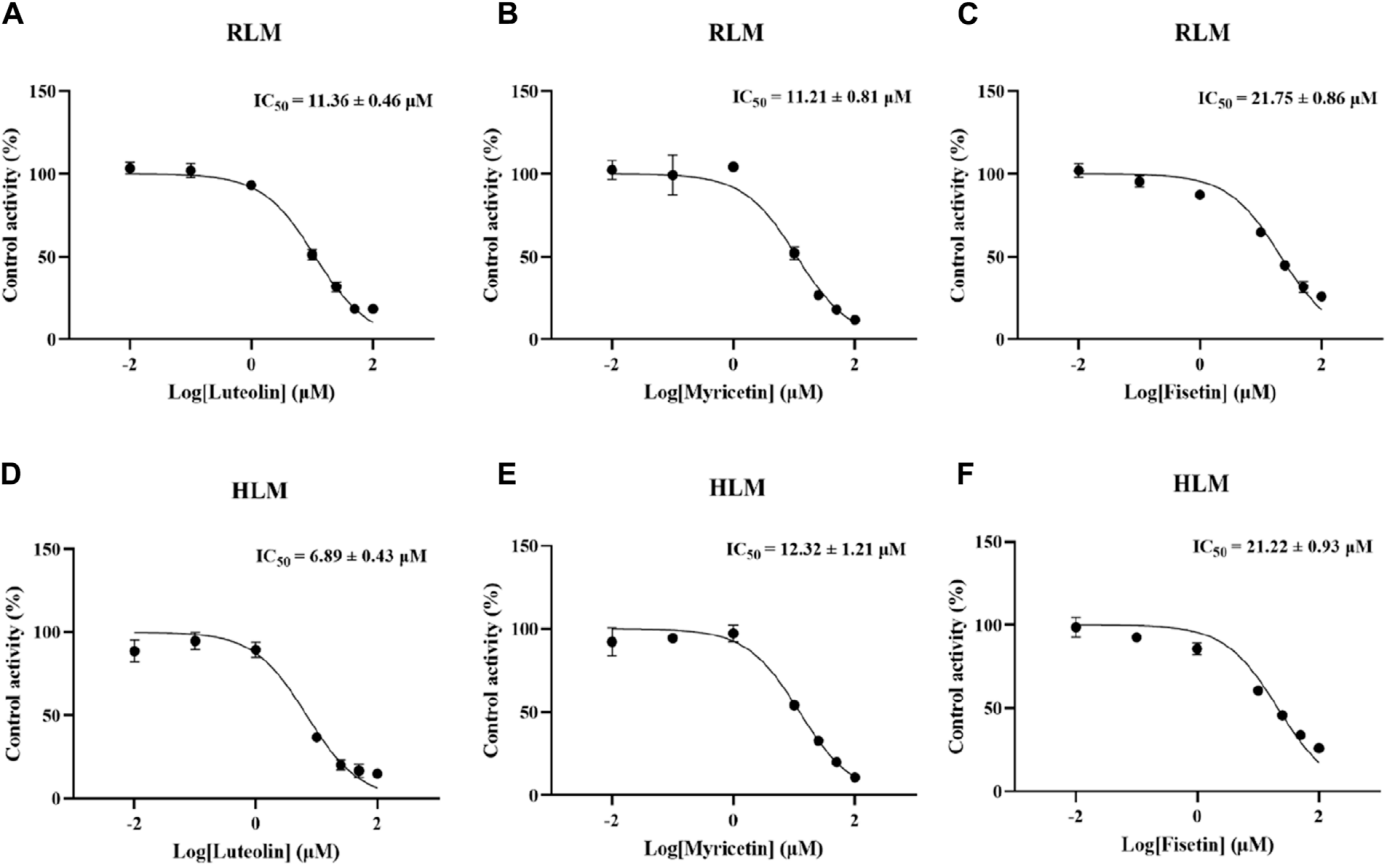
Inhibitory potency of three kinds of hepatoprotective TCM on lenvatinib metabolism *in vitro*. **(A**–**C)** The half-maximal inhibitory concentration (IC_50_) of luteolin **(A)**, myricetin **(B)** and fisetin **(C)** in RLM, respectively. **(D**–**F)** The IC_50_ curve diagram of luteolin **(D)**, myricetin **(E)** and fisetin **(F)** in HLM. Data are presented as the mean ± S.D.

According to Figure 2, in RLM, the IC_50_ of luteolin, myricetin, and fisetin to inhibit the metabolism of lenvatinib were 11.36 ± 0.46 µM, 11.21 ± 0.81 µM, and 21.75 ± 0.86 µM, respectively, which showed that luteolin and myricetin can mightily reduce the metabolic activity of lenvatinib. Additionally, in HLM, the IC_50_ values of luteolin, myricetin, and fisetin were 6.89 ± 0.43 µM, 12.32 ± 1.21 µM, and 21.22 ± 0.93 µM, respectively, in which the inhibition rate of luteolin was significantly higher than in RLM.

### 3.3 Effects of three flavonoids on CYP1A2, CYP2B6, and CYP3A4 activity in RLM

**FIGURE 3:**
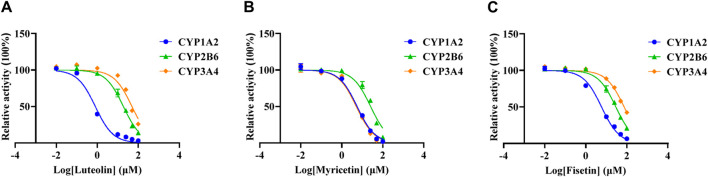
Effects of three flavonoids on CYP1A2, CYP2B6 and CYP3A4 activity in RLM. The half-maximal inhibitory concentration (IC_50_) of luteolin **(A)**, myricetin **(B)** and fisetin **(C)**.

The K_m_ values of the three specific CYP isoform probes (melatonin, bupropion and midazolam) were determined by the mixed method to be 32.39 ± 0.58 μM, 8.30 ± 0.52 μM and 1.90 ± 0.13 μM, respectively. The IC_50_ values of three flavonoids were determined according to the mixed K_m_ values, as shown in Figure 3 and Table 2. When the concentration of luteolin was 100 μM, it showed strong inhibition on CYP1A2, where the activity of the enzyme was inhibited by 96.89% ± 0.23%. In addition, luteolin demonstrated moderate inhibition on CYP2B6 and weak inhibition on CYP3A4. Similar inhibition degree of myricetin was observed on CYP1A2 (IC_50_ = 5.61 ± 0.25 µM) and CYP3A4 (IC_50_ = 5.11 ± 0.11 µM), while it exhibited an IC_50_ value of >20 µM on CYP2B6. The degree of inhibition of fisetin on CYP1A2 and CYP2B6 were similar to that of myricetin, and it showed weak inhibition on CYP3A4.

**TABLE 2:** Enzymatic kinetic parameters (K_m_ and IC_50_) for CYP1A2, CYP2B6 and CYP3A4 of three flavonoids in RLM.

Enzymes	K_m_ (μM)	IC_50_ of luteolin (μM)	IC_50_ of myricetin (μM)	IC_50_ of fisetin (μM)
CYP1A2	32.39 ± 0.58	0.81 ± 0.07	5.61 ± 0.25	5.89 ± 0.13
CYP2B6	8.30 ± 0.52	18.92 ± 1.33	25.61 ± 1.73	29.42 ± 1.36
CYP3A4	1.90 ± 0.13	49.43 ± 2.45	5.11 ± 0.11	74.24 ± 2.16

### 3.4 Metabolic stability

**FIGURE 4:**
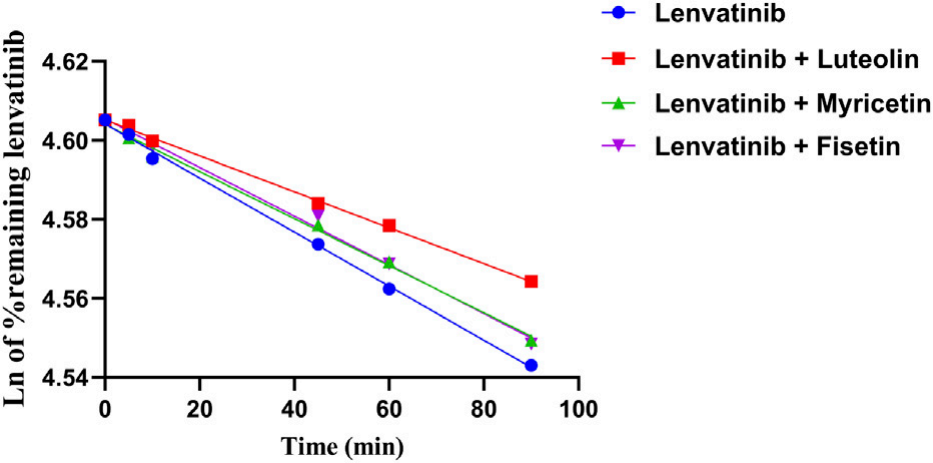
Linear part of the metabolic stability curve of lenvatinib and three flavonoids; n = 3.

The relative content of lenvatinib in the RLM matrix was calculated after stopping the metabolic reaction at different time periods. The relative content was equal to the percentage of the remaining lenvatinib relative to zero time (representing 100%). As shown in Figure 4, the natural logarithm of the percentage of remaining lenvatinib and incubation time were linearly regression, then the slope k was obtained. The *in vitro* half-life (t_1/2_) was obtained using the equation: t_1/2_ = 0.693/k; V (mL/mg) = volume of incubation (Kaci et al., 2023)/protein in the incubation (Rumgay et al., 2022); intrinsic clearance (CL_int_) (mL/min/mg protein) = V × 0.693/t_1/2_. In RLM, the t_1/2_ of lenvatinib was 1018.12 ± 93.22 min, and CL_int_ was 0.0023 ± 0.0002 mL/min/mg. The metabolic stability results of three flavonoids co-incubated with lenvatinib, including t_1/2_ and clearance were shown in Table 3. The results suggested that under the action of three flavonoids, metabolism of lenvatinib slowed down and CL_int_ decreased *in vitro*, which verified the results of pharmacokinetic experiments.

**TABLE 3:** Linear regression equation for the linear portion of t_1/2_ and CL_int_ in the presence or absence of three flavonoids.

Groups	Linear regression equation	t_1/2_	CL_int_ (mL/min/mg)
Lenvatinib	Y = −0.0006843*X + 4.604	1018.12 ± 93.22	0.0023 ± 0.0002
Lenvatinib + Luteolin	Y = −0.0004550*X + 4.605	1526.18 ± 105.08	0.0015 ± 0.0001
Lenvatinib + Myricetin	Y = −0.0005956*X + 4.604	1134.29 ± 128.73	0.0021 ± 0.0004
Lenvatinib + Fisetin	Y = −0.0006165*X + 4.605	1067.29 ± 111.00	0.0022 ± 0.0002

### 3.5 Luteolin increased the drug exposure of lenvatinib in SD rats

**FIGURE 5:**
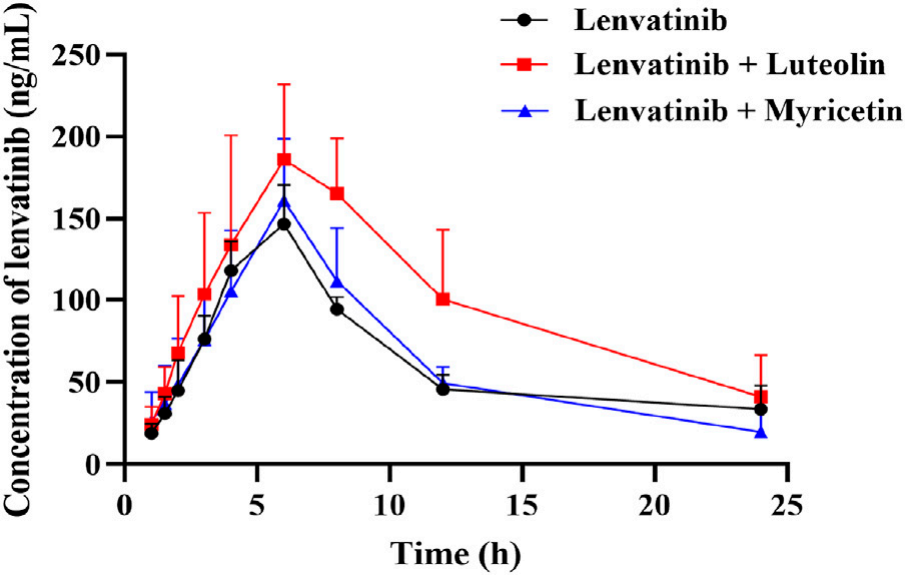
Mean plasma concentration-time curve of lenvatinib in three groups. Data are presented as the mean ± S.D.; n = 5.

The mean plasma concentration-time curves of lenvatinib in different groups were shown in Figure 5. The results indicated that the combination of luteolin with lenvatinib increased plasma exposure to lenvatinib, and from the results of the main pharmacokinetic parameters in Table 4, luteolin could rise the AUC_(0-t)_ and AUC_(0-∞)_ of lenvatinib by 0.81 and 1.18 times, respectively, while also reducing the elimination rate of lenvatinib by 0.49-fold, thus increasing the accumulation or prolonging the residence time of the drug in SD rats. However, there was no significant difference when lenvatinib and myricetin were administered together compared to single-use.

**TABLE 4:** Main pharmacokinetic parameters of lenvatinib in three groups (n = 5).

Parameters	Lenvatinib	Lenvatinib + luteolin	Lenvatinib + myricetin
AUC_(0-t)_ (ng/mL*h)	1,258.79 ± 304.44	2,274.90 ± 730.45*	1,479.94 ± 218.75
AUC_(0-∞)_ (ng/mL*h)	1,412.02 ± 206.62	3,079.66 ± 1,177.36*	1,549.61 ± 241.20
t_1/2z_ (h)	4.14 ± 1.57	11.15 ± 6.85	4.90 ± 2.20
T_max_ (h)	5.60 ± 0.89	5.00 ± 1.41	6.00 ± 0.00
CL_z/F_ (L/h/kg)	0.86 ± 0.12	0.44 ± 0.17**	0.79 ± 0.12
C_max_ (ng/mL)	146.68 ± 20.45	183.45 ± 40.45	160.86 ± 37.77

Notes: AUC, area under the blood concentration–time curve; t_1/2z_, elimination half time; T_max_, peak time; CL_z/F_, blood clearance; C_max_, maximum blood concentration. *p < 0.05, **p < 0.01, compared with the singel group.

### 3.6 Luteolin inhibited lenvatinib metabolism by different mechanisms in RLM and HLM

**FIGURE 6:**
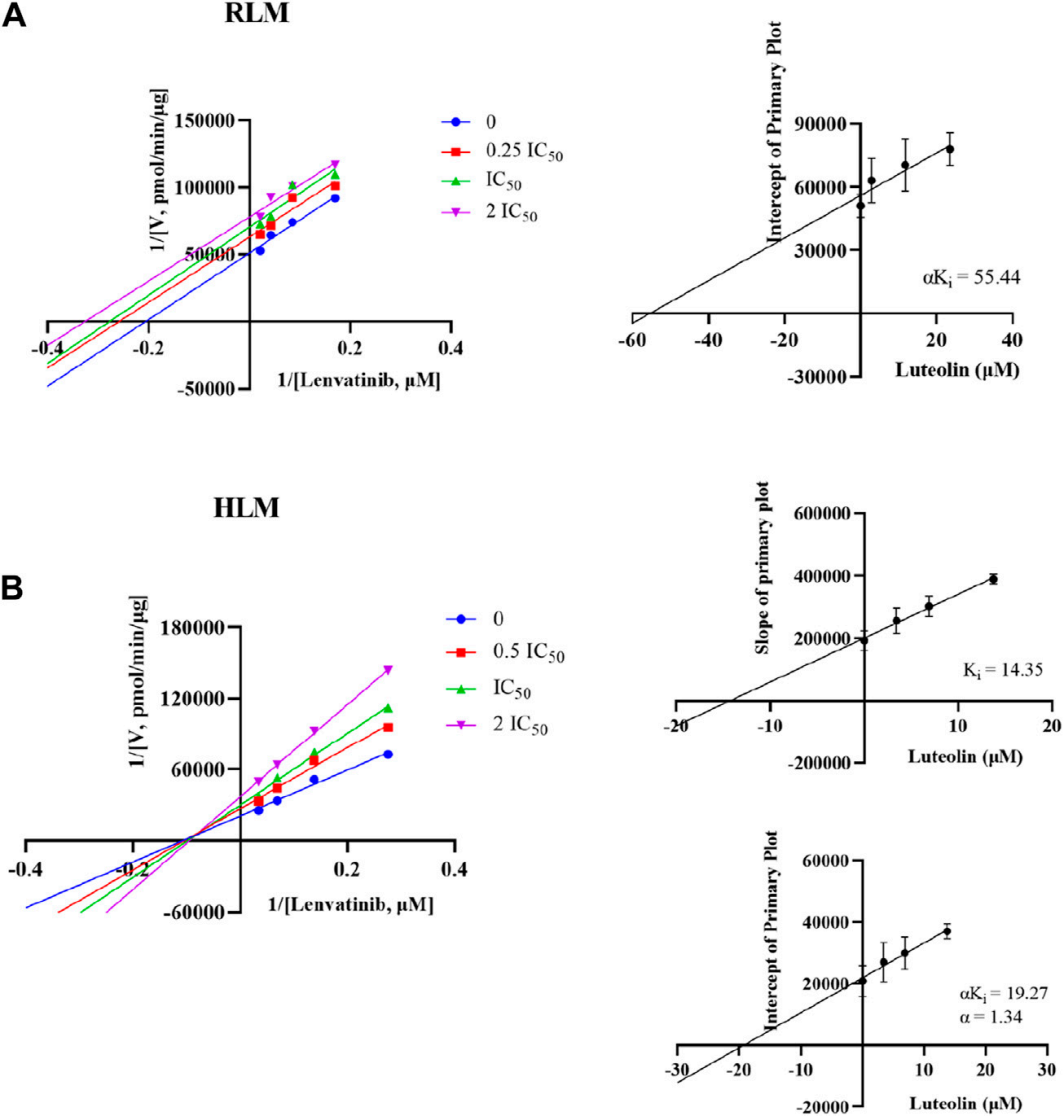
Potential inhibitory mechanism of luteolin on lenvatinib. **(A)** Lineweaver-Burk double reciprocal plot and its secondary plot for αK_i_ of luteolin inhibiting lenvatinib metabolism in RLM. **(B)** Lineweaver-Burk double reciprocal plot, its secondary plot for αK_i_, and its secondary plot for K_i_ of luteolin inhibiting lenvatinib metabolism in HLM. Data are presented as the mean ± S.D.; n = 3.

The Michaelis-Menten constant K_m_ of lenvatinib was 23.5 ± 2.17 µM in RLM, while K_m_ was 16.9 ± 2.42 µM in HLM. Interestingly, Figure 6 showed the different mechanism types of luteolin inhibiting lenvatinib metabolism in RLM and HLM. Luteolin was an un-competitive inhibition in RLM, with a αK_i_ = 55.44 ± 17.73 µM, while in HLM, it presented a mixed type of non-competitive and competitive inhibition, with K_i_ = 14.35 ± 4.85 μM and αK_i_ = 19.27 ± 4.16 μM, respectively.

## 4 Discussion

HCC is one of the most common malignancies worldwide and the leading cause of cancer-related death (Vogel et al., 2022). Its prognosis is poor and the mortality rate (830,000 deaths per year) is almost similar to the worldwide incidence (Mazzanti et al., 2016; McGlynn et al., 2021; Siegel et al., 2022). Lenvatinib is a multi-target TKI with anti-VEGFR 1–3, FGFR 1–4, PDGF, and KIT (Tohyama et al., 2014). According to a Phase III REFLECT study (Kudo et al., 2018), lenvatinib treatment is not inferior to sorafenib, which is the first first-line targeted drug approved for the treatment of HCC (Galle et al., 2021). In addition, EMA and FDA have now also approved lenvatinib for the first-line treatment of HCC (Vogel et al., 2021). However, lenvatinib generally appears with hepatotoxicity, palmar-plantar erythrodysesthesia syndrome, proteinuria, and other adverse reactions during treatment (Furuse et al., 2023). To greatly improve the overall survival rate of patients, the combination of drugs with hepatoprotective agents is necessary.

The anti-tumor effect of TCM has been widely recognized (Zhao et al., 2023), which exhibit lower toxic side effects than chemotherapy drugs, and are usually taken as food additives or dietary supplements, greatly improves the possibility of interaction between TCM and drugs (Kotwal et al., 2020). Among them, flavonoids commonly found in our daily life have anti-oxidation, anti-cancer, liver protection, and other biological activities (Li et al., 2018). In this study, we systematically chose luteolin, myricetin, and fisetin, to explore their impacts on lenvatinib metabolism both *in vitro* and *in vivo*.

Results of the *in vitro* experiment showed that one of the enzyme kinetic parameters, K_m_, in RLM was 23.5 µM, while the K_m_ value in HLM was 16.9 µM, which was 28.1% lower than in RLM. This was mainly due to the fact that CYP3A4 is widely expressed in HLM, while the CYP2C subfamily is its main enzyme in RLM (Rendic and Di Carlo, 1997; Indra et al., 2019), so there was a stronger affinity between lenvatinib and HLM. In RLM, the IC_50_ of luteolin, myricetin, and fisetin for lenvatinib was 11.36 ± 0.46 µM, 11.21 ± 0.81 µM, and 21.75 ± 0.86 µM, respectively. Among them, both luteolin and myricetin had potentially inhibitory effects on lenvatinib metabolism. At the same time, the results of our study on the inhibition degree of three flavonoids on specific CYP isoform probes (CYP1A2, 2B6 and 3A4) also confirmed that luteolin and myricetin were potential inhibitors of CYP1A2. In RLM, when the inhibitory concentration was 100 μM, the inhibitory rates of luteolin, myricetin and fisetin on CYP1A2 were 96.89% ± 0.23%、98.12% ± 0.19% and 93.56% ± 0.22%, respectively. Besides, the IC_50_ values of luteolin, myricetin, and fisetin in HLM were 6.89 ± 0.43 µM, 12.32 ± 1.21 µM, and 21.22 ± 0.93 µM, respectively. Particularly, luteolin may be more likely to increase the risk of adverse reactions of lenvatinib in humans than in SD rats.

*In vivo* experiments, when luteolin was combined with lenvatinib, the results were in agreement with the conclusions *in vitro* and can observably increase the AUC_(0-t)_ and AUC_(0-∞)_ of lenvatinib by 0.81-fold and 1.18-fold in SD rats, respectively. However, it is slightly less likely to expand toxic side effects than ketoconazole or isavuconazole, which can improve plasma exposure to lenvatinib by 3.01-times or 50.20% (Xia et al., 2023). Nevertheless, the pharmacokinetic parameters of lenvatinib did not change when myricetin and lenvatinib were administered together, which may be due to the poor stability of myricetin in the gastrointestinal tract, and eventually reduce its bioavailability and validity (Xiang et al., 2017). Previous studies had shown that the low absolute bioavailability of myricetin in rats (less than 10%) was attributed to its poor water solubility, which may also be the reason why myricetin had no inhibitory effect on lenvatinib in rats (Hong et al., 2014).

The enzyme responsible for lenvatinib O-demethylation are CYP1A1, 1A2, 2B6, and 3A4 (Vavrová et al., 2022). Furafylline (CYP1A2 selective inhibitor) had a 37% inhibitory effect on the formation of *O*-desmethyl lenvatinib, which is consistent with the study of Liu et al. (Vavrová et al., 2022; Liu et al., 2023). In previous studies, flavonoids were shown to have strong inhibitory effects on CYP2C8 and CYP1A2 (Kaci et al., 2023). This was also confirmed in our experiment, where the IC_50_ values of the three flavonoids against CYP1A2 were all <10 μM. Luteolin is a potent CYP1A1 inhibitor and has been reported to inhibit CYP1A2 *in vitro* with IC_50_ values < 10 μM (Cao et al., 2017; Shi et al., 2024). Myricetin has been shown to inhibit CYP3A4 and CYP3A2 in RLM and HLM (Lou et al., 2019). In one study, myricetin inhibited tofacitinib non-competitively in both RLM and HLM, with IC_50_ values of 9.27 μM and 2.35 μM, respectively (Ye et al., 2024), consistent with *in vitro* results from our experiment. *In vitro* metabolic stability experiments also revealed that luteolin and myricetin had inhibitory effects on lenvatinib metabolism, and the clearance was decreased to a similar degree.

## 5 Conclusion

Lenvatinib may have the possibility to be combined with flavonoids, especially luteolin, that can stratify the efficacy of lenvatinib. Thus, when using lenvatinib and flavonoids together in clinical practice, special attention should be paid to avoiding the interaction with luteolin.

## Data Availability

The original contributions presented in the study are included in the article/Supplementary Material, further inquiries can be directed to the corresponding authors.
